# Study protocol for a prospective observational study to investigate the role of luminal pressure on arteriovenous fistula maturation

**DOI:** 10.1097/MD.0000000000017238

**Published:** 2019-10-04

**Authors:** Ho-Shun Cheng, Te-I Chang, Cheng-Hsien Chen, Shih-Chang Hsu, Hui-Ling Hsieh, Chun-You Chen, Wen-Cheng Huang, Yuh-Mou Sue, Feng-Yen Lin, Chun-Ming Shih, Jaw-Wen Chen, Shing-Jong Lin, Po-Hsun Huang, Chung-Te Liu

**Affiliations:** a Division of Cardiology, Department of Internal Medicine, Wan Fang Hospital; b Department of Surgery, School of Medicine, College of Medicine; c Division of Cardiovascular Surgery, Department of Surgery, Wan Fang Hospital, Taipei Medical University; d Graduate Institute of Biomedical Electronics and Bioinformatics, National Taiwan University; e Division of Nephrology, Department of Internal Medicine, Wan Fang Hospital; f Division of Nephrology, Department of Internal Medicine, Shuang Ho Hospital; g Department of Internal Medicine, School of Medicine, College of Medicine; h Emergency Department, Department of Emergency and Critical Medicine, Wan Fang Hospital; i Department of Emergency Medicine, School of Medicine, College of Medicine, Taipei Medical University; j Graduate Institute of Medical Science, National Defense Medical Center; k Department of Radiation Oncology, Wan Fang Hospital; l Graduate Institute of Clinical Medicine, College of Medicine, Taipei Medical University; m Division of Cardiology and Cardiovascular Research Center, Department of Internal Medicine, Taipei Medical University Hospital; n Division of Cardiology, Department of Medicine, Taipei Veterans General Hospital; o Cardiovascular Research Center, National Yang-Ming University; p Department of Medical Research, Taipei Veterans General Hospital; q Institute of Pharmacology ^r^ Institute of Clinical Medicine, National Yang-Ming University; s Board of Directors, Taipei Medical University, Taipei, Taiwan.

**Keywords:** arteriovenous fistula (AVF), end-stage renal disease, hemodialysis, luminal pressure, vascular access

## Abstract

**Introduction:**

Arteriovenous fistula (AVF) is the preferred vascular access for hemodialysis due to its higher patency and lower infection rate. However, its suboptimal maturation rate is a major weakness. Although substantial risk factors for AVF maturation failure have been disclosed, modifiable risk factors remain unknown. During the AVF maturation process, an elevated luminal pressure is required for outward remodeling; however, excessively high luminal pressure may also be detrimental to AVF maturation, which remains to be defined. We hypothesized that higher AVF luminal pressure is harmful to its maturation, and investigate its potential as a modifiable factor to improve AVF maturation.

**Methods and analysis:**

This prospective study includes patients undergoing surgical creation for a native AVF. The exclusion criteria were as follows: age <20 years, inability to sign an informed consent, and failure to create a native AVF due to technical difficulties. Demographic and laboratory profiles will be collected before AVF surgery. Vascular sonography will be performed within 1 week of AVF creation to measure the diameters, flow rates, and flow volumes of AVF and its branched veins. The pressure gradient within AVF will be estimated from the blood flow rates using the modified Bernoulli equation. The primary outcome is spontaneous AVF maturation defined as provision of sufficient blood flow for hemodialysis within 2 months of its creation without any interventional procedures. The secondary outcome is assisted AVF maturation, which is defined as AVF maturation within 2 months from its creation aided by any interventional procedure before the successful use of AVF.

**Discussion:**

While contemporary theory for AVF maturation failure focuses on disturbed wall shear stress, complicate assumptions and measurement preclude its clinical applicability. AVF luminal pressure, which may be manipulated pharmaceutically and surgically, may be a target to improve the outcome of AVF maturation.

**Trial registration:**

This study has been registered at the protocol registration and results system. The Protocol ID: NCT04017806.

## 1 Introduction

In Taiwan, the burden of end-stage renal disease has been increasing tremendously over the last two decades. According to the 2017 Annual Report on Kidney Disease in Taiwan, the prevalence of patients requiring dialysis has increased from 1379 per million population in 2000 to 3317 per million population in 2015, thereby demonstrating an increasing rate of 11.1% per year.^[1]^ In patients on dialysis treatment, vascular access dysfunction is the most common cause of medical procedures, including angioplasty, thrombectomy, and placement of central venous catheters, thereby indicating a need for improved medical care associated with vascular access.^[2]^

Arteriovenous fistula (AVF) was introduced as a vascular access for hemodialysis by Brescia et al^[3]^ and remains the most commonly used vascular access to date.^[4]^ Due to its superior access patency and lower infection rates, AVF is the preferred vascular access for maintenance hemodialysis.^[5]^ However, AVF is not an ideal vascular access without drawbacks. One of its major flaws is that only 50% to 80% of the created AVFs achieve functional maturation for hemodialysis,^[6–8]^ which results in infectious and thromboembolic complications due to the prolonged use of the central venous catheter.

Previous studies have revealed several risk factors of AVF maturation failure, including anemia, advanced age, diabetes mellitus, and smoking.^[9,10]^ While these risk factors may be targeted to improve AVF maturation rate, modifiable risk factors for prevention are currently lacking.^[9]^ On the other hand, basic studies have revealed molecular pathogenic factors of AVF maturation failure, including localized inflammation, hypoxic injury, oscillating wall shear stress, uremic milieu, and oxidative stress.^[11]^ Despite these substantial studies exploring the role of pathogenic factors, modifiable pathogenic factors that are applicable for improvements in AVF maturation are lacking.^[12]^

Maturation failure of AVF results from luminal stenosis due to neointimal hyperplasia, signifying a thickening of the subintimal area caused by proliferation of myofibroblasts as shown by remarkable staining for α-smooth muscle actin and vimentin.^[13]^ Neointimal hyperplasia of AVF occurs mainly at the arteriovenous junction and venous limb of AVF, where the venous endothelium is exposed to a non-physiological high blood flow rate, oscillatory shear stress, and pulsatile stretching strain by arterial blood flow, suggesting the role of altered hemodynamics on AVF subintimal proliferation.^[14]^

Physically, the human arterial and venous hemodynamics are substantially different. In the arterial system, the blood flow is pulsatile with a mean arterial pressure of 65 to 70 mmHg, while in venous system, the blood flow is non-pulsatile with a blood pressure of 5 to 10 mmHg.^[15]^ Furthermore, endothelial cells from the arterial and venous parts of the circulation system have different genetic expression profiles, indicating that different hemodynamic environments regulate phenotypes of endothelial cells.^[16]^ Therefore, it is reasonable that elevated luminal pressure may stimulate proliferation in the venous endothelium of AVF and result in maturation failure. In contrast, elevated AVF luminal pressure also provides a stretching force for outward remodeling, which is required for AVF maturation. As such, an optimal range of luminal pressure needs to be defined.

Consequently, we hypothesized that while elevated AVF luminal pressure is required for AVF maturation, excessive AVF luminal pressure may cause a detrimental stretching force and result in AVF maturation failure. However, an optimal luminal pressure range remains to be defined.

## 2 Methods and analysis

### 2.1 Main aims

The aim of this study will be conducted to investigate the optimal AVF luminal pressure for AVF maturation, which may be modified surgically or pharmaceutically to improve the AVF maturation rate.

#### 2.1.1 Specific aims

**Aim 1: To characterize the altered blood flow rates and luminal pressures in different segments of AVF**

While excessive AVF luminal pressure may stimulate the proliferation of AVF venous subintima to cause maturation failure, comprehensive AVF hemodynamic profiles in different AVF segments should be characterized first. In this study, the blood flow rates will be measured at the artery-to-vein anastomosis, the bifurcation sites of AVF, and its branches after bifurcation. With these flow rate values, the pressure gradient across the AVF will be calculated. We will also estimate the absolute AVF luminal pressure using the difference between the mean arterial pressure and AVF pressure gradient. These results will provide a comprehensive hemodynamic profile of AVF and may reveal novel and valuable risk factors of AVF maturation failure.

**Aim 2: To characterize the association between luminal pressures and the vessel diameters of different segments of AVF**

Previous studies have shown that a cephalic venous diameter <2.5 mm independently predicted AVF maturation failure.^[17]^ However, the association between the vessel diameters and AVF hemodynamics is complex, and the concept that a larger vessel diameter provides higher maturation rate may be inappropriate. For example, a cephalic vein with a larger diameter may also have larger branches to offset the AVF luminal pressure and outward remodeling required for AVF maturation. In this part of the study, the association between the vessel diameters of different segments and AVF luminal pressure will be analyzed. The results will provide preliminarily evidence to support the role of vessel diameter in AVF maturation.

**Aim 3: To investigate the association between AVF luminal pressure and AVF maturation rate**

The pressure gradient and absolute luminal pressure in AVF will be calculated using the blood flow rates in different sites of the AVF. With these results, the association between pressure values and AVF maturation rates will be analyzed. These results may improve our understanding about the role of AVF luminal pressure at maturation. Moreover, valuable and modifiable risk factors for AVF maturation failure may be disclosed, which may be manipulable surgically or pharmaceutically in future.

**Aim 4: To investigate the association between AVF luminal pressure and known risk factors of AVF maturation failure**

As described above, the suggested risk factors of AVF maturation failure include anemia, advanced age, diabetes mellitus, and smoking.^[18]^ Nonetheless, how these risk factors actually affect AVF hemodynamics remained to be defined. In the present study, these known risk factors of AVF maturation failure and laboratory profiles associated with renal failure will be collected at the time of the AVF creation. The association between calculated luminal pressure and these risk factors will be analyzed. These results will provide novel evidence to support the pathogenic role of the known risk factors of AVF maturation failure.

### 2.2 Study design and procedure

This prospective observational study is mainly aimed to investigate the association between AVF luminal pressure and maturation rate. Patients who meet the following eligibility criteria are eligible for enrollment:

1. patient at pre- or post-dialysis status who undergoes surgical creation of native AVF for hemodialysis, and
2. AVF created at both radial and brachial arteries.

### 2.3 Participants

In the present study, patients scheduled to have AVF will be informed about current limitations of AVF technique and the need to improve AVF maturation rate. Then, we explain how this study will help patients receiving AVF creation in the future and encourage them to participate the study voluntarily. Patients who decide to participate the study will be explaining the time required to complete the measurements. Apart from the above statement, patients/the public were not involved in the study design, development of the outcomes and measurements, or dissemination of the study results.

#### 2.3.1 Inclusion criteria

**Figure 1:**
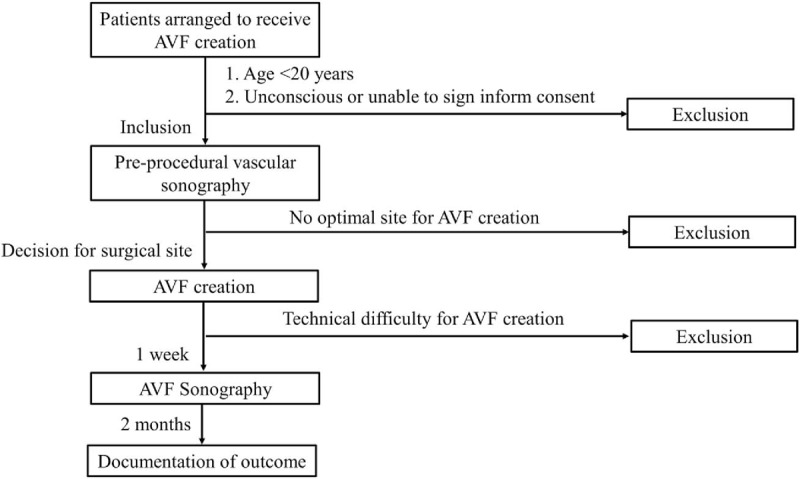
Flowchart of the study design. AVF = arteriovenous fistula.

The included participants will receive vascular sonography to measure the diameters, flow rates, and flow volumes of AVF and its branched veins within 1 week from the surgical creation of AVF (Fig. 1).

#### 2.3.2 Exclusion criteria

The exclusion criteria are as follows:

1. patients <20 years of age,
2. patients who are unconscious or unable to sign the informed consent,
3. patient in whom native AVF creation is shifted to arteriovenous graft placement due to technical difficulty.

#### 2.3.3 Recruitment

Subject recruitment began in September 2018 at Wan Fang Hospital, Taipei Medical University, Taipei, Taiwan and is expected to end by May 2020. The recruitment of participants, collection of parameters, and confirmation of the outcomes will be performed by a fulltime study nurse.

### 2.4 Variables

#### 2.4.1 Demographic variables

The demographic profiles of the included patients, including gender, age, body mass index, smoking status, and culprit disease of renal failure will be documented. Comorbidities including diabetes mellitus, hypertension, coronary artery disease, peripheral arterial occlusion disease, atrial fibrillation, and related medication will be recorded.

#### 2.4.2 Laboratory analysis

The laboratory data of pre-surgical evaluation, including hemoglobin and white blood cell count as well as levels of serum urea, creatinine, sodium, potassium, calcium, phosphorus, albumin, and parathyroid hormone will be included as covariates. Before the creation of AVF, pre-procedural vascular sonography of both arms will be performed to determine the optimal site for the AVF. After the creation of the AVF, vascular sonography of the ipsilateral arm will be repeated within 1 week to measure the diameters, blood flow rates, and blood flow volume of the AVF and its main branches as described previously. The AVF luminal pressure will be calculated from blood flow rates using the modified Bernoulli equation as described in the following section.

#### 2.4.3 Estimation of luminal pressure of AVF

For the radiocephalic AVF, the blood flow rates will be measured at the radial artery-to-cephalic vein anastomosis and the site of cephalic vein bifurcation into the basilic and cephalic veins. The luminal pressure in the radiocephalic AVF will be estimated by the pressure gradient from these two points. For the brachiobasilic AVF, blood flow rates will be measured at the brachial artery-to-basilic vein anastomosis and basilic vein-to-brachial vein junction. The luminal pressure of the brachiobasilic AVF will be estimated by the pressure gradient between these two points. For the brachiocephalic AVF, the blood flow rates will be measured at the brachial artery-to-median basilic vein anastomsosis and cephalic vein-to-subclavian vein junction. The luminal pressure of the brachiocephalic AVF will be estimated by a pressure gradient between these two points.

The blood flow rates of the AVF and its branches will be measured by Doppler ultrasound (Zonare Ultrasound System, Shenzhen Mindray Bio-Medical Electronics Co.). All Doppler ultrasound examinations will be performed by 2 rotating technicians to ensure consistency between measurements. Then, the pressure gradient between the arterial and venous ends of the AVF will be estimated using the modified Bernoulli equation as following: 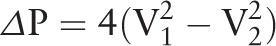


Where ΔP refers to the pressure gradient, V_1_ refers to the proximal blood flow rate, and V_2_ refers to the distal blood flow rate. The absolute blood pressure of the arterial limb of the AVF will be approximated as the mean arterial pressure of the contralateral arm. Then, the blood pressure of the AVF will be approximated with the following equation: 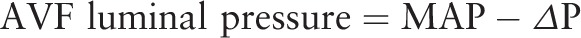


Where MAP refers to the mean arterial pressure measured in the contralateral brachial artery.

### 2.5 Outcomes

The primary outcome of this study is spontaneous AVF maturation, which is defined as provision of sufficient blood flow for hemodialysis within 2 months of its creation without any interventional procedures. The secondary outcome is assisted AVF mature, which is defined as AVF maturation within 2 months from its creation, which is aided by any interventional procedure before successful use of AVF. The maturation time is defined as the period from the date of AVF creation to the date of the first successful employment for hemodialysis.

### 2.6 Statistical plan

Continuous variables will be expressed as mean ± standard deviation, while nominal variables are expressed in frequency and percentage. Comparisons of continuous variables will be performed using the 2-tailed *t* test for unpaired samples or Welch *t* test as appropriate. Comparisons of nominal variables will be performed using the Chi-square test or Fisher exact test as appropriate. Multivariate logistic regression test will be used to evaluate the association between predictor variables and outcome variables. Statistical significance will be defined by a *P* value of <.05. The statistics will be performed using SAS 9.4 (SAS Institute Inc, Cary, NC).

G∗Power 3.1.9.4 was used to estimate the sample size to reach statistical significance in the *t* test. Assuming the effect size to be 0.6. Under the condition that α error is defined as 0.05; power was defined as 0.8; and the allocation ratio was defined as 1. Therefore, the sample size required to achieve statistical significance is 90 patients.

The data used for the study will be preserved and analyzed by the primary investigator. The data is accessible only to the primary investigator and study nurse for data safety. The data will be preserved for 2 years after the end of the study.

### 2.7 Ethics and dissemination

This study has been approved by the ethics committee and Institutional Review Board of Taipei Medical University (N201801091). All participants will sign the informed consent after being informed about the goals and methods of the study. The present study will be conducted in accordance with the tenets of the 1975 *Declaration of Helsinki*, as revised in 2000. The result of the study will be disseminated by publication as journal articles.

## 3 Discussion

The contemporary theory for AVF maturation failure or loss of long-term AVF patency focuses on disturbed wall shear stress. With the creation of AVF, arterial blood flow is introduced into the venous system, raising the venous wall shear stress from 1–6 dyn/cm^2^ to 10–70 dyn/cm^2^, similar to the level in the arterial system. Because of the increased wall shear stress, the vein initiates outward remodeling, a process that expands vessel diameter and lowers wall shear stress towards its baseline level, forming the AVF for hemodialysis.^[19]^ For blood flow in a vessel, wall shear stress is estimated by the Haagen-Poisseuille equation as follows: 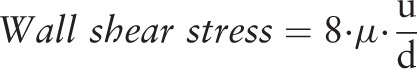


where **μ** is the kinetic viscosity, **u** is the mean velocity, and **d** is the vessel diameter. Note that this equation can only be applied in vivo with the assumptions that blood is considered as a Newtonian fluid; the blood vessel is cylindrical, straight, and inelastic; and that blood flow is steady and laminar.^[20]^ The role of disturbed wall shear stress on AVF stenosis has been demonstrated in animal^[21]^ and computational^[22]^ models of AVF. Recently, shear stress has also been considered to be a potential key factor for AVF maturation.^[23]^ However, blood wall shear stress estimations requires complicated hemodynamic measurements and several assumptions that are difficult to be applied to human subjects, thereby making it hard to standardize wall shear stress in clinical studies.^[24]^ In the present study, we expect to demonstrate the association between AVF luminal pressure and AVF maturation, which may be modified pharmaceutically or surgically.

## Author contributions

**Conceptualization:** Ho-Shun Cheng, Te-I Chang, Shih-Chang Hsu, Hui-Ling Hsieh, Chun-You Chen, Chung-Te Liu.

**Data curation:** Shih-Chang Hsu, Hui-Ling Hsieh, Chun-You Chen, Chung-Te Liu.

**Formal analysis:** Shih-Chang Hsu.

**Investigation:** Te-I Chang, Hui-Ling Hsieh, Chung-Te Liu.

**Methodology:** Chung-Te Liu.

**Project administration:** Hui-Ling Hsieh, Chung-Te Liu.

**Supervision:** Ho-Shun Cheng, Te-I Chang, Cheng-Hsien Chen, Wen-Cheng Huang, Yuh-Mou Sue, Feng-Yen Lin, Chun-Ming Shih, Jaw-Wen Chen, Shing-Jong Lin, Po-Hsun Huang, Chung-Te Liu.

**Writing – original draft:** Chung-Te Liu.

**Writing – review & editing:** Chung-Te Liu.
